# "My wife's mistrust. That's the saddest part of being a diabetic": A qualitative study of sexual well-being in men with Type 2 diabetes in sub-Saharan Africa

**DOI:** 10.1371/journal.pone.0202413

**Published:** 2018-09-10

**Authors:** Sara Cooper, Natalie Leon, Hazel Namadingo, Kirsten Bobrow, Andrew J. Farmer

**Affiliations:** 1 Cochrane South Africa, South African Medical Research Council, Cape Town, South Africa; 2 Division of Social & Behavioural Sciences, School of Public Health and Family Medicine, University of Cape Town, Cape Town, South Africa; 3 Health Systems Research Unit, South African Medical Research Council, Cape Town, South Africa; 4 Malawi Epidemiology Intervention Research Unit, Lilongwe, Malawi; 5 Nuffield Department of Primary Care Health Sciences, University of Oxford, Oxford, United Kingdom; 6 Chronic Disease Initiative for Africa, Department of Medicine, University of Cape Town, Cape Town, South Africa; Emory University, UNITED STATES

## Abstract

**Introduction:**

Sexual dysfunction is a common complication for men with diabetes, yet little is known about the lived experiences of sexual difficulties within the context of diabetes, particularly in low-and-middle-income countries. This study explores how men with type 2 diabetes in three sub-Saharan African settings (Cape Town and Johannesburg, South Africa; Lilongwe, Malawi) perceive and experience sexual functioning and sexual well-being, and the biopsychosocial contexts in which these occur and are shaped.

**Methods:**

We used a qualitative research design, including individual interviews (n = 15) and focus group discussions (n = 4). Forty-seven men were included in the study. We used an inductive thematic analysis approach to develop our findings. A biopsychosocial conceptual model on the relationship between chronic illness and sexuality informed the interpretation of findings.

**Results:**

Men across the study settings identified sexual difficulties as a central concern of living with diabetes. These difficulties went beyond biomedical issues of erectile dysfunction, comprising complex psychological and relational effects. Low self-esteem, related to a sense of loss of masculinity and reduced sexual and emotional intimacy in partner relationships were common experiences. Specific negative relational effects included suspicion of infidelity, mutual mistrust, general unhappiness, and fear of losing support from partners. These effects may impact on men’s ability to cope with their diabetes. Further stressors were a lack of information about the reasons for their sexual difficulties, perceived lack of support from healthcare providers and an inability to communicate with partners about sexual difficulties.

**Conclusion:**

More in-depth research is needed to better understand sexual functioning and well-being within the context of diabetes, and its potential impact on diabetes self-management. Holistic and patient-centered care should include raising awareness of sexual problems as a potential complication of diabetes amongst patients, their partners and care providers, and incorporating sexual well-being as part of routine clinical care.

## Introduction

There is growing recognition that sexual dysfunction is a common and potentially serious complication of diabetes in men and women globally [1, 2]. Studies in high income countries (HICs) have found diabetes to be a significant risk factor for erectile dysfunction (ED), with men with diabetes reported to experience a three-times greater age-adjusted probability of ED than those without diabetes [3]. Similar trends have been found in sub-Saharan Africa (SSA). For example, studies in Ghana [4], Nigeria [5] and Ethiopia [6] have observed prevalence rates of ED in men with diabetes to be between 68% and 73%, while a study in South Africa found a two-times increased rate of ED in patients with diabetes, compared to men without diabetes [7]. Erectile dysfunction has also been shown to be a strong predictor of poor quality of life and negative psychosocial outcomes among men with diabetes, including in SSA [8, 9].

Studies on the high prevalence, risk and burden of sexual dysfunction in diabetes, especially amongst men, has been instrumental in raising the profile of this issue. Yet sexual dysfunction amongst people with diabetes is still neglected within clinical practice. As noted in a recent review of global diabetes guidelines, “sexual dysfunction is still an under-appreciated, under-recognized, under-discussed, and commonly untreated complication of diabetes” [2]. Moreover, little is known about how people with diabetes perceive and experience sexual difficulties, and the bio-psychosocial contexts of these experiences, particularly in low- and middle-income countries (LMICs) [10–12]. These issues are difficult to identify and explore through checklists of physiologic sexual dysfunction and quality of life scales typically used in quantitative research on sexual functioning [11, 13]. Yet qualitative social science research on this topic is scarce, particularly in LMICs [13, 14]. With the exception of research in Brazil [15, 16], most qualitative studies on sexuality in the context of diabetes have been conducted in HICs, including the USA [17], UK [11], Sweden [18], and the Netherlands [14]. This small body of research in HICs has provided important insights into the negative psychological effects of sexual difficulties for patients and their partners, and the adverse impact of these difficulties on patients’ gender identities and interpersonal relationships. However, more in-depth qualitative explorations in this area, which investigate broader notions of sexual wellbeing and personhood beyond bio-medical sexual dysfunction, is needed and especially in LMICs [10].

The aim of this paper is to report on the perceptions and subjective experiences of sexual functioning and sexual well-being amongst men with diabetes in three SSA settings—Cape Town and Johannesburg, South Africa and Lilongwe, Malawi.

Studies have shown that the relationship between diabetes and sexual dysfunction is unclear and likely to be multifactorial, including neurologic, vascular, endocrine and psychosocial mechanisms related to both the disease and treatment [19, 20]. Verschuren et al [10] offer a biopsychosocial conceptual model on the relationship between chronic illness and sexuality that acknowledges the complex influences on the experience of sexual functioning. Verschuren and colleagues developed this model through a comprehensive review of the literature as well as their many years of clinical experience. The model has subsequently been used as a guiding theoretical framework in various studies on chronic illness and sexuality [12, 21, 22]. The key assumption of this model is that sexuality is a multifaceted phenomenon, shaped by organic, hormonal, and psychosocial factors, and that chronic illness involves psychosocial stressors in addition to physical symptoms. The model distinguishes “sexual functioning” from “sexual well-being”, with the former comprising the bio-medically defined ‘normal’ physiological standards of the sexual response cycle and the latter involving the subjective experience of sexuality in the context of an individual’s personal life and relational situation.

This multidimensional model and its key assumptions thus provides a useful theory-oriented framework for understanding the various interacting biopsychosocial processes that shape perceptions and experiences of sexual functioning and sexual well-being within the context of diabetes. We incorporated this model during the analysis stage of our research to enrich our interpretation of the findings. That is, we used it to help us understand the relationship between the biomedical and social experiences of sexuality that we saw emerging from the data, and to make a distinction between experiences of sexual functioning and sexual well-being. A better understanding of these issues will provide important insights on the lived experience of sexual difficulties, and associated impact, meanings, values and priorities amongst men with diabetes. This in turn can be used to inform strategies to better respond to the sexual concerns and support needs of people with diabetes, ultimately contributing to more effective, comprehensive, and holistic treatment and care.

## Methodology

### Study design

This is a qualitative study using focus group discussions (FGDs) and individual interviews (INTs) for data collection. The data on sexuality is a secondary analysis of data collected for a broader formative and pre-trial process evaluation study of a randomized controlled trial investigating the impact of a Short Message Service (SMS) text message intervention to support diabetes treatment adherence in SSA- *the StAR2D Study* (Trial registration number: ISRCTN70768808). The formative and process evaluation study explored the lived experiences and perspectives of diabetes and diabetes care, as well as access and attitudes towards mobile phones as a health promotion tool.

We chose to use both in-depth interviews and focus group discussions as combining these methods can facilitate a deeper comparison of perspectives, improve data completeness and enhance the trustworthiness of findings [23]. While interviews enable one to explore individual views and experiences in detail, the conversational nature and interaction in a focus group can help generate additional insights, test and refine understandings gained in individual interviews, and stimulate commentary that may not have been elicited from individual interviews [24]. Ultimately, triangulating the data through using both these methods can strengthen the credibility and reliability of qualitative research findings.

Ethical approval to conduct the study was obtained from the University of Cape Town (126/2015), University of Witwatersrand (R14/49), National Health Sciences Research Committee of Malawi (15/7/1425) and Oxford Tropical Research Ethics Committee (22–15). All participants who were included in the study provided written, informed consent to participate.

### Participants and recruitment

Participants in the formative and process evaluation study were men and women, 18 years and older, clinically diagnosed with type 2 diabetes and taking diabetes treatment (oral or both oral and insulin). Recruitment occurred at urban public healthcare facilities in Cape Town, Johannesburg and Lilongwe in Malawi. These were the trial sites chosen for a comparison of the trial intervention across three SSA settings. These areas have a high burden of type 2 diabetes, and patients receive healthcare and medication from their local healthcare facility. Care is provided free of charge, including essential medicines at no cost to patients. Settings differ in context in terms of the organisation of health care services, level of resources and degree of urbanisation of the patient population (with the most urbanised population being in Johannesburg).

Purposive sampling was used to ensure representation of the wide range of patients with type 2 diabetes in these three areas with regards to age, gender, ethnicity, language, and duration of diabetes. Study fieldworkers informed adults with type 2 diabetes about the study and handed out information leaflets while they were waiting for care at the public health care clinics. Individuals who expressed interest in the study were asked to contact the study personnel using contact details provided on the information leaflets or to share their phone numbers to set up an appointment.

### Data collection

This sub-analysis on sexuality draws on the data obtained from the FGDs and individual interviews conducted with men in all three settings. The composition of FGDs for the main study were stratified by gender (i.e. men only and female only groups), to enable participants to potentially talk more freely about their personal views and experiences. All the male participants who took part in the broader formative and process evaluation study (including those who were interviewed and those who took part in the male FGDs) constitute the sample for this secondary analysis on sexuality (See Table 1 for Demographic details).

**Table 1:** Characteristics of participants (N = 47N.

Participants	Cape Town	11 (1 FGD, 8 INTs)
	Johannesburg	15 (1 FGD)
	Lilongwe	21 (2 FGDs, 7 INTs)
Age (years)	< 40	4
	40–49	11
	50–59	12
	60–70	19
	>70	1
Type of diabetes treatment	Oral medication only	38
	Oral medication and insulin	9
Time with diabetes (years)	<5	27
	5–10	12
	>10	8

FGDs lasted 2–3 hours, while interviews lasted between 1 and 2 hours. Interviews and FGDs were conducted by researchers trained in qualitative research, and took place between November 2015 and July 2017. In Malawi, data collection was done in Chichewa, and in South Africa, data collection was done predominantly in English, and with translation assistance in Afrikaans and isiXhosa (Cape Town) and isiZulu (Johannesburg). Using semi-structured topic guides, the following themes were explored in FGDs and interviews: basic demographics; experiences and perceptions of diabetes; sources of information and support; challenges and opportunities in managing diabetes; experiences of receiving healthcare; and views about an SMS text message intervention to provide support for diabetes (S1 Appendix. Interview and Focus Group Topic Guide). Whilst the interview and FGD guides did not focus on sexuality, male and a few female participants raised the issue spontaneously when asked about their experiences of living with diabetes. Male participants raised the issue of sexuality more commonly, more consistently and in greater depth. This provided material for a secondary analysis of their experience of sexuality in relation to living with diabetes. In the few cases (3 interviews) where sexuality was not spontaneously raised by the participants themselves, we probed by asking: *“Sometimes people living with diabetes find it hard to be intimate with their partners*. *Is this something you experience*?*”*. Interviews and FGDs were digitally recorded, transcribed and translated into English where applicable.

### Data analysis

The data was analysed through an inductive approach [25] as the topic of sexuality emerged out of a broader data set on the lived experience of diabetes, and without predetermined questions. After each interview and FGD, the interviewers wrote detailed summaries. We reviewed and reflected on these summaries as they became available, and this formed the initial level of iterative data analysis. Using these post-interview summaries, together with an initial reading of the full transcripts, SC, NL, HN first created a list of conceptual components (‘opening coding’) regarding experiences of living with diabetes and receiving healthcare. SC and HN then independently coded transcripts through line-by-line readings and with the aid of *Nvivo*10 (in South Africa) and *MaxQDA12* (in Malawi). Inter-coder checking revealed that the use of different data management software did not impact on coding validity. Additional codes were developed iteratively, agreed upon amongst the three researchers (SC, HN, NL) and added to the coding framework.

Data or sections related to sexuality were then labelled according to the codes in the coding framework, such as ‘information’; ‘effect- physical’; ‘effect-emotional’; ‘effect-relational’; ‘context’; ‘source of support’. Once the data was labeled with main codes, we used a thematic analysis approach [26] to categorize, extract and collate the data into themes. For each theme, we paid attention to the meaning given to the issue, as well as the subjective, socio-cultural and relational contexts in which it was situated. Further discussions between SC, NL, HN were then undertaken to check if these themes ‘fitted’ with the coded extracts and overall data-set; refine the specifics of each theme; understand the relationships between the sexuality themes; and contextualize these themes within other pertinent topics emerging out of the data. During the analysis, we searched for a conceptual framework about sexuality and chronic disease. We found the framework developed by Verschuren et al [10] to be a useful tool to help us understand the relationship between the biomedical and social experiences of sexuality that we saw emerging from the data. The conceptual framework helped us to organize our findings into the two main categories—sexual functioning and sexual well-being- and to enrich our interpretation of the findings.

## Results

### Demographics

In total, 47 participants were included in the study: 11 from Cape Town, 15 from Johannesburg and 21 from Lilongwe. In-depth individual interviews were conducted with 15 male participants (7 in Lilongwe and 8 in Cape Town) while 32 participants in total took part in focus group discussions (2 FGDs in Lilongwe, and 1 each in Johannesburg and Cape Town). The mean number of FGD participants was 8, with the smallest group in Cape Town (3 participants) and the largest group in Johannesburg (15 participants). Mean age of all participants was 55.3 years (SD: 11.2). The youngest participant was 28 years old; the oldest 78 years. All FGDs comprised a mix of ages: ages ranged from 28–69 years in the Johannesburg FGD; 45–65 years in the Cape Town FGD; and 32–70 years in one FGD in Lilongwe and 41–68 years in the other Lilongwe FGD. Nearly half (48.9%) of participants had been living with diabetes for between 1 and 5 years and hypertension was the most common co-morbid condition. Most participants (80.9%) were taking oral medication only for their diabetes (See Table 1).

### Sexual functioning

#### Sexual difficulties a key concern of living with diabetes

When asked about their general experiences of living with diabetes, men across all three settings and in both focus groups and interviews spontaneously identified sexual difficulties as a key issue. They often raised the topic at the beginning of the focus group or interview, but several also returned to the topic throughout the discussion, demonstrating the importance of the issue for them. The high level of interest in this topic is illustrated by an interaction that took place at the start of one of the FGDs. The facilitator opened the discussion by asking about what daily life with diabetes involves, and the following emotionally-charged exchange ensued:

*Respondent 1: The biggest problem we have as men is when we go to the bedroom…There's only one real problem, and every man has this…It's weakness in the bedroom*.

*Respondent 2: Absolutely. We don’t perform*.

*Respondent 3: Nothing happens for me at night…I can’t do anything*.

Respondent 4: Yes. Every man with diabetes can differentiate the way he was playing his bedroom game before diabetes and then after getting the disease…

*Respondent 2*: *I mean*, *yes*, *we are old…But we should be able to manage 1 round*, *maybe once a week*. *But not once a year* [laughs]

*Respondent 5: Yeah, it doesn’t matter if it goes for a long or short time*. *But as long as I’ve*

*done something…But I can’t even do that*.

                                                                                                    *[FGD2, Site C]*

This exchange characterises the nature of subsequent discussions about sexuality in focus groups, as well as in interviews. Talk about sexuality was unprompted and sexual dysfunction was presented as a pressing and key concern of living with diabetes. Although there was acknowledgment that older age is involved, the men conveyed a strong sense that diabetes was responsible in one way or another for their sexual dysfunction. Finally, they expressed the desire to continue being sexually active (even if in a limited way), and were distressed that their basic sexual functioning was curtailed. Similar complaints were expressed in different ways by others, with the common sentiment being the difficulty to perform sexually and associated intimacy problems, for example, feeling that they failed to perform, feeling *“completely dead”* in the bedroom, and on an emotional level, feeling that there is no happiness in the bedroom.

As seen above, sexual difficulties were most commonly spoken of euphemistically as *“bedroom problems”* and *“body weakness”*. Specific physiological symptoms mentioned included: reduced sexual desire, reduced sexual arousal and an inability to sustain an erection or ejaculate, which limited sexual intercourse and sexual intimacy.

#### Need for better understanding of the causes of sexual difficulties

Although most men attributed their sexual problems to diabetes in one way or another, there was considerable uncertainty about why and how this came about. When asked directly what they knew about the reasons for their sexual difficulties, they either had no idea or were uncertain or confused. All men said they wanted to know more about the possible reasons for their sexual dysfunction. Frequently the men wondered about whether it was the diabetes disease itself or the diabetes medication (or its side-effects) that caused their sexual difficulties:

*I don’t know if it’s because of the diabetes itself, or if it’s the meds, or what. Maybe it’s something else. I just don’t get it*.

                                                                                                    *[INT15*, *Site A]*

This uncertainty and need for more information was common across all three settings. When probed about their sense of how diabetes may be involved, responses differed. Participants commonly referred to indirect notions of diabetes *“weakening”* the body. For some, more than just *“weakening”* the body, diabetes and/or diabetes medication was perceived as causing physiological damage to the body and sexual organs by, for example, producing a blockage in the sexual organs.

### Sexual well-being

Experiences of sexual dysfunction seemed to go beyond the bio-medical. The men elaborated on the psychological and interpersonal effects of experiencing sexual dysfunction. Low self-esteem, related to a sense of loss of masculinity, and reduced intimacy in partner relationships were common experiences. A sense of insecurity in the relationship, mistrust from partners and fear of abandonment were some of the relational effects that negatively affected sexual well-being.

#### Loss of manhood

Not being able to live up to their own or their partner’s expectations of earlier levels of sexual performance affected the men’s self-image, especially their sense of manhood. A range of sentiments were expressed related to feeling powerless as a man and *“doomed”* in performing their male sexual role. Insecurity about their male identity made them feel that their role in the partnership was diminished. A participant summed up a shared sentiment about this changing dynamic:

*We are not real men anymore. We have become children again or else a friend of our wives*.

                                                                                                    *[FGD2*, *Site C]*

In several reports, the inability to perform sexually, and consequently the inability to sexually satisfy one’s partner, was experienced as virtually synonymous with a loss of one’s manhood.

#### Reduced emotional intimacy and relationship quality

Several participants reported that their female partners had expressed unhappiness about their sexual performance. For some, this unhappiness spilled over into discord in the couple’s relationship in general, including ongoing quarrelling and conflict, strained relationships and a general sense of unhappiness in the home. The men were unable to provide their partner with satisfactory reasons for their change in sexual performance. Mistrust, blame and being falsely accused of infidelity were commonly reported responses from their partners. Some women reportedly felt that the sexual difficulties signaled a lack of romantic interest in them, and some had suspicions or accused the men of seeking sexual liaisons outside of their relationship. A participant explained being at the receiving end of such accusations:

*It’s causing problems for me and my wife. She thinks I’m having sex with other women…When my wife sees my performance she says things like*: *‘Why you so weak? Have been bewitched by another lady? Did you have sex during the day? You think I’m a fool?’…*

                                                                                                    *[INT11*, *Site A]*

The level of mistrust and damage to emotional intimacy was experienced as a heavy burden. A participant shared his distress regarding the negative effects on his relationship:

*She thinks it’s my fault I can’t do it properly. She thinks I’m seeing other women…And really, it breaks my heart …that she’s become so mistrustful and is pushing me away because of this…If I was sleeping around, or being a bad husband, then I could understand my wife’s treatment…But it’s not my fault…I wish my wife could see that and not accuse*.

                                                                                                    *[INT2*, *Site B]*

Participants described the damage to their emotional intimacy as being extremely sad and hurtful. For some, the associated stress potentially threatened their ability to cope with diabetes. A participant tearfully described his ongoing distress about his wife leaving the marital bedroom, and his worry about the effects of this stress on his health:

*I worry about it all the time…Always thinking about it, stressing, what I can do to make things right. It’s probably making me more sick, all this stressing*. *I’m just miserable because of this…*

                                                                                                    *[INT7*, *Site B]*

Across the three settings, the negative psychological and relational effects associated with their sexual difficulties were described as the most disturbing part of living with diabetes. This sentiment is aptly captured in the words of one participant:

*“My wife’s mistrust. That’s the saddest part of being a diabetic”*.

                                                                                                    *[INT4*, *Site B]*

Mistrust did not only come from female partners, but also from the men themselves, who feared that their partner might seek sexual satisfaction elsewhere. Some men expressed more dire fears—that their partner will abandon them altogether due to dissatisfaction with the relationship. They valued the emotional and social support they received from partners to help them cope with their diabetes. Many men provided narratives about how their female partners cook them healthy meals, encourage them to exercise and remind them to take their medication regularly. Losing their partner would thus also mean losing this central source of support for coping with diabetes. A participant explained:

*I worry she might look elsewhere to satisfy her needs…I worry about it every day because I don’t know how I’d cope without her. Really. She helps me with everything. Does everything to take care of me*.

                                                                                                    *[INT3*, *Site B]*

### Support for sexual functioning and sexual well-being

#### Not feeling supported by healthcare professionals

Despite the long-standing nature of sexual difficulties for most, participants spoke of not having received the information, advice and support they needed to cope with these. Many frequently referred to their continued need for help from healthcare providers. When asked directly if they had raised the issue with healthcare providers, responses varied. Most had not raised it, and the few who had, were unsatisfied with the responses they received. They reported that healthcare providers had never asked about, or offered any information on sexual functioning at routine follow-up visits. Moreover, there was never discussion about sexual dysfunction being a possible complication of diabetes in health-awareness talks.

Reasons for not raising the topic with healthcare providers included embarrassment and feeling *“awkward”* or *“uncomfortable”* about discussing the issue. Other participants felt there was a lack of capacity amongst health care workers to deal with such issues and/or that staff were too overburdened and consultation times too short to discuss sexual difficulties. A more common reason, mainly amongst South African participants, was concern about bad treatment by health care providers, including feeling scared of being scolded, blamed or shamed. A participant explained the fear of speaking to a health care provider:

*I don’t say anything. You know how they are at the clinic…They shout at you when you ask things. Tell you to stop complaining. And sometimes even punish you for wasting time…So, I’m scared to speak to the doctor*.

                                                                                                    *[FGD2*, *Site C]*

The few who had managed to raise the topic reported that they had received unhelpful and even hurtful responses that were experienced as dismissive and even punitive, including blaming and shaming, as illustrated here:

*I’ve shared my problem with them. But…the nurse, she just said ‘Look at your health. You know why the boy down there won't work. You don’t look after yourself. If you started to take care of your body, your problem would go away*.

                                                                                                    *[FGD2*, *Site C]*

Healthcare providers reportedly often dismissed their concerns by saying that sexual dysfunction was normal with ageing and that the men should just accept this. By contrast, participants felt that they needed continued intimacy with their partners, despite their age and disease status, but that health care providers dismissed and ridiculed this need.

Participants reported a few instances where health care workers tried to reassure them, for instance by encouraging them not to worry so much and to move on from this concern. One doctor warned that stressing about sexual problems may worsen the man’s diabetes. From the perspective of the participants, none of those attempts were experienced as helpful. A few men reported receiving a prescription for libido-enhancing drugs to help them improve their performance in bed, but this had unpleasant side-effects that were hard to cope with, including palpitations, dizziness and other “*scary effects”*. Lack of affordability was another obstacle, as these drugs are costly and not available in the public sector.

#### A silent and unsolvable problem?

As shown earlier, relational effects of sexual difficulties amongst men include mutual mistrust, conflict and unhappiness, relationship insecurity and even fear of losing one’s partner. Yet, the men most commonly refrained from discussing sexual difficulties with their partner. They felt the topic was too awkward or difficult to discuss and/or that they did not know how to communicate about it. A few participants indicated that talking about it would only make things worse, and thus perceived avoiding the topic to be the best option.

In the few cases when it was raised, the women reportedly were unable and/or unwilling to talk about it, changing the subject or not engaging when there was an opening to discuss it. A participant explained how his light-hearted attempt to discuss the topic did not bear fruit:

*I have spoken to my wife. I tried to make a light thing of it…I joked ‘I’ll ask the doctor for pills to make me stiff for you’. But, she didn’t say anything…I could tell she didn’t want to talk about it*.

                                                                                                    *[INT2*, *Site B]*

Although participants expressed the desire to resolve their sexual difficulties, most could not see a clear solution. Expressions of pessimism and a sense of desperation and despair were common, as were expressions of their ongoing need for help with the problem. One participant expressed a sense of resignation, feeling that nothing could be done about his sexual difficulties:

*I’ve come to realise that I must just accept it ‘cause nothing can be done. No-one can help me*.

                                                                                                    *[INT1*, *Site B]*

Another man remained hopeful for a sudden improvement in his sexual performance and the sexual well-being of his partnership:

*“I pray every day…that one day I’ll wake my wife up and say ‘let’s do it’ and I’ll be able to. And she’ll once again see me as the man she married”*.

                                                                                                    *[INT6*, *Site B]*

Across the three settings, there was no reference to seeking health care advice with one’s partner, except for one participant. This participant described how, following persistent accusations about infidelity, he suggested that he and his partner seek help from their clinic. A healthcare provider educated the couple about sexual dysfunction being a complication of diabetes, and this helped them to adjust their relationship, despite his continuing erectile dysfunction.

*I tried to talk to her but she just accused me of seeing another woman…So I suggested we go for counselling…When we went she got information about diabetes, including complications like impotence…Since then understands better and now we live happily despite sometimes I cannot perform as well as she wants*.

                                                                                                    *[INT15*, *Site A]*

## Discussion

In this study we report on how men with type 2 diabetes in three SSA settings (Cape Town and Johannesburg, South Africa and Lilongwe, Malawi) perceive and experience sexual functioning and well-being, and the biopsychosocial contexts of these experiences. We used Verschuren et. al.’s [10] conceptual model on the association between chronic disease and sexuality to make a distinction between experiences of sexual functioning and sexual well-being, and to understand the processes by which disease-related psychological and relational factors may, interactively, affect the sexual lives of men with diabetes. To our knowledge, this is the first qualitative study in SSA to focus on men’s perceptions and experience of sexuality in the context of diabetes.

The men in this study identified sexual problems as a fundamental concern of living with diabetes. Most reported experiencing significant changes in sexual functioning since being diagnosed with diabetes; including a loss of or decrease in sexual interest, an inability to have or maintain an erection as well as a more general sense of having ‘problems’ or ‘unhappiness’ in the bedroom. Difficulties with sexual desire, erectile function, and ejaculation are indeed commonly reported as direct and indirect consequences of the disease amongst men with diabetes [10]. What was striking was the gravity of the psychological and interpersonal effects of these sexual problems. That is, the men’s experiences of sexual dysfunction went beyond the biomedical, impacting on their gender identity, emotional intimacy with partners and sense of security in the relationship. These experiences and perceptions were remarkably similar amongst the participants and across the three study settings.

A sense of failed gender identity emerged as a particularly significant psychological consequence of the men’s sexual difficulties. An inability to live up to expectations of sexual performance was perceived as a form of emasculation. In Brazil, studies with men living with diabetes had similar findings, where sexual problems negatively affected the men’s sense of manhood [15, 16]. These sentiments may be connected to prevailing socio-cultural notions of masculinity. In many societies, including in LMICs, virility and potency is linked to the ‘successful’ enactment of masculinity, and thus a man’s inability to ‘properly’ perform sexually is often perceived as a sign of weakness and vulnerability [27–29]. In line with these ideals, men in this study interpreted their sexual difficulties as ultimately a loss of manhood, which affected their self-worth and their sense of security in their intimate relationships.

Strained and conflictual intimate relationships was another major effect of the men’s experiences of sexual dysfunction. The relationship between chronic illnesses and ‘partner relationship’ is emphasized in the Verschuren et. al’s [10] conceptual model: chronic disease can influence a couple’s sexual relationship through both the physical and psychological effects the illness has on the well-being of the patient, and on their partner. Qualitative studies in HICs have also highlighted the negative reciprocal effects of sexual problems on intimate relationships [14, 17, 18].

Verschuren and colleagues [10] highlight the need for a better understanding of the interactive mechanisms through which a chronic disease may affect a couple’s sexual well-being and quality of their relationship. In this regard, a key finding in our study was the central role of mistrust and insecurity in the relationship. The couple’s sexual well-being was negatively affected by the diminished sexual activity, but also by the gendered psychological and social reactions of the men and their partners, which included a sense of emasculation, suspicion of infidelity, blame and conflict, mutual mistrust, and a general sense of unhappiness in the relationship.

There were indications that these psychosocial effects may impact on the men’s ability to manage their diabetes, a concern that is also acknowledged in Verschuren et. al’s conceptual model [10]. Some men feared that the loss of sexual and emotional intimacy may lead to more serious emotional estrangement, and ultimately abandonment. A few spoke directly about how their high levels of stress threatened their ability to cope with diabetes, while others feared that they could lose the critical social support that their partners provide. Fears about the potential negative effects of losing a partner’s social support may not be unfounded. Studies have shown that social support, including from one’s intimate partner, has a significant effect on the self-management of diabetes [30–32], including improving treatment adherence and associated glycemic control [33], as well as enhancing self-care activities [34, 35].

Despite the pervasiveness and negative effects of the men’s sexual problems, and despite their expressed need for more information, there appears to be a persistent silence on the part of the men themselves, their partners, and their health care providers. This is due to a combination of factors: a lack of knowledge or confusion about the reason for their sexual difficulties, an inability and/or unwillingness to communicate with one’s partner, and a general sense of unresponsiveness from healthcare workers. Other qualitative studies have also identified a lack of awareness and uncertainty about the reasons for sexual difficulties amongst patients with diabetes [11, 14–16].

The perceived lack of support from healthcare professionals was another key concern of participants. In the experience of these men, no healthcare provider had ever initiated a conversation about their intimate lives, nor provided them with any information on sexual dysfunction as a possible complication of diabetes. At the same time, the men found it difficult to raise the issue themselves, either out of embarrassment or fear of being ridiculed (a fear born out in the experience of some participants) or, alternatively, because they believed that healthcare workers lack the capacity and/or time to respond appropriately. Studies in HICs have similarly found that men with diabetes are often reluctant to talk to health care professionals because of anxiety, fears of rejection and a lack of time during consultations [36–40]. This non-discussion between professionals and patients has been described as a “conspiracy of silence”, in which both parties know sexuality is a pertinent issue, but for a variety of reasons, are unwilling or unable to talk about it [41].

What was particularly striking was the accounts of unhelpful, sometimes hurtful, and even punitive responses from healthcare providers, particularly in South Africa. Even efforts to reassure patients came across as patronizing and dismissive, only further discouraging the men from seeking help from healthcare workers. These experiences of negative treatment by health care providers may be part of a more general unresponsive health care environment [42, 43]. For example, participants in an earlier study on hypertension in Cape Town reported similar concerns about a general lack of responsiveness on the part of health care providers and punitive approaches to patients who were considered to be ‘non-adherent’ to hypertension treatment [44].

Sexual health is considered a basic human right and a fundamental part of leading a full and healthy life for many people [45], including those with chronic illnesses [21, 46]. Moreover, and as detailed in Verschuren et al.’s conceptual model [10], problems of sexual functioning and sexual well-being may be a barrier to effective self-management of diabetes, yet we need a better understanding of the mechanisms through which this may occur. In our study, we show the potential mechanisms through which sexual difficulties may be threatening self-management, be it through chronic and high levels of stress, through fractured intimate relationships, and/or through fear of losing social support from partners (real or perceived)- the latter being directly related to the ability to self-manage diabetes.

To promote more comprehensive, patient-centered, and effective diabetes care, healthcare workers may want to consider the value of increasing patient awareness of sexual problems as a complication of diabetes. This information could go some way towards helping patients to better understand the underlying reasons for their sexual difficulties and for them to find ways to address it constructively, including gaining the understanding and support of their partners. It would be useful for healthcare workers to be aware of the multiple ways in which sexual difficulties may be experienced and it’s emotional and relational impact. Healthcare workers should understand that, while aging might bring about changes in sexuality, reduced sexual functioning is by no means an inevitable process of aging [12, 47]. To assist healthcare practitioners, more concrete diabetes care guidelines which address sexual dysfunction and well-being, and which are currently limited [2], need to be developed. However, these strategies will most likely only be successful if they are part of broader, more responsive and patient-centered healthcare service, whereby patients are actively supported in their efforts towards self-management and staff are supported and motivated to provide comprehensive diabetes care.

### Strengths and limitations

We took several steps to improve the methodological rigor of the research. Triangulating the use of individual interviews and focus groups helped to strengthen the credibility and reliability of the findings [23]. We also considered the importance of enabling participants to engage in their first language: in Malawi data collection was entirely in Chichewe and in South Africa, where people are more bilingual, we used translators and encouraged participants to use their language of preference. As we had not set out to explore sexuality, we did not consider whether the female gender of researchers would be a barrier to male participant engagement on this topic. Our experience was that the men seized the opportunity to raise sexual difficulties as a key concern and that our female gender did not seem to limit their spontaneity and level of engagement.

Other steps taken to improve credibility included triangulation in the data analysis phase (independent analysis by 2 researchers, SC & HN, in the first phase) and consultation amongst the social scientists from South Africa and Malawi (SC, NL, HN) through two face-to-face workshops. At the early stage, we developed a code book based on our discussion of the emerging findings; we reached consensus on codes and themes which enhanced the inter-coder reliability of the analysis.

Our study has several limitations. Two of the FGDs discussions deviated from methodological recommendations of appropriate focus group size. We aimed for the standard size recommended for a focus group (i.e. between 6 to 10 participants) and invited more participants, assuming there would be no-shows [24]. For one group few (only 3) participants arrived, whilst for another group, all invited participants (15) arrived. Moderating the conversation in a too small or a too large group is more difficult and can negatively affect the productivity of the group (as not all may engage fully in the discussion) [24]. The variation in group size for these two FGDs thus may have negatively affected the depth and richness of the data.

Further, we did not set out to study the issue of sexuality, with a predetermined set of questions or framework, and this poses several limitations. The study did not probe issues that could have provided further depth of understanding. Verschuren et al. [10] detail a range of issues pertaining to sexuality and chronic disease which we were unable to explore in this study, such as the influence of age, the form of diabetes treatment and the duration of living with diabetes. As mentioned, we were struck by the similarity of experiences amongst the men in this study, but further probing may have revealed greater heterogeneity based on the above or other factors, such as, for example, socioeconomic and relationship contexts. These issues would benefit from further study, especially in LMICs, where the burden of chronic disease is growing rapidly.

We also did not stratify the FGDs by age, and thus the groups ended-up comprising men from a large range of age groups. This mix of ages may have influenced the group dynamics and interactions, and thus shaped what some felt comfortable and able to talk about (or not). Given that age has been found to impact on the relationship between sexuality and chronic disease [10], further research which targets and distinguishes between different age groups would be useful.

Our analysis also focuses only on men, a gender-bias which reflects data that emerged out of the broader study. Had we asked participants directly about sexuality, and probed more deeply, it may have emerged as a key issue for women in our study. We thus need more in-depth qualitative research amongst women about the effects of their own and/or their partner’s sexual difficulties in the context of living with diabetes.

Furthermore, we used Verschuren et al. [10] conceptual model at the end stage of our study, thus limiting the value that such a framework could have added to the design and data collection stage of the study. Future studies of sexuality and chronic illness may benefit from consulting this or other frameworks at the design stage of the study.

Finally, while the study found striking similarities in accounts across the three different SSA sites, the men in our study all live in or close to urban settings and were recruited from public sector health care facilities providing free diabetes care. The transferability of these findings to similar or different settings therefore requires careful judgement [25].

## Conclusion

Sexual difficulties emerged as a key and pressing concern for men with diabetes in this study. These difficulties went beyond physiological impediment to intercourse, comprising complex psychological and relational meanings and effects. There is a need for greater awareness on the part of patients, their partners, and healthcare workers of the experiences of sexual difficulties in the context of living with diabetes, and increased efforts to address this constructively. More in-depth research, amongst both men and women, is needed to better understand the importance of sexual functioning and sexual well-being in supporting diabetes self-management, especially in light of the rising prevalence of diabetes in LMIC settings.

## Supporting information

S1 Appendix Interview and Focus Group Topic Guide. (DOCX)
