# Supplementary material for: "My wife's mistrust. That's the saddest part of being a diabetic": A qualitative study of sexual well-being in men with Type 2 diabetes in sub-Saharan Africa
Source: PLoS One. 2018 Sep 10;13(9):e0202413. doi: 10.1371/journal.pone.0202413 (PMC6130865; doi:10.1371/journal.pone.0202413)
Supplement: S1 Appendix — (DOCX) [file pone.0202413.s001.docx]

**S1 Appendix: Focus Group and Interview Topic Guide**

**Welcome/introduction**

Thank you for agreeing to take part in this interview/focus group discussion. Before I begin I would like to explain again why we would like to talk to you. For this interview, we want to hear your views and experiences of living with diabetes to better understand what it’s like to live with a long- term disease and to know more about your experience of your health care. We would also like to understand how you currently use your mobile phone and your views on receiving messages about health care. (For pre-trial process evaluation, individual interviews only): We would also like to hear about expectations of participating in the *StAR2D study. All of this will help us better understand the opinions and experiences of the people who are participating in the *StAR2D study.)

It is important to emphasise that there are no right or wrong answers. We want to hear about your views and experiences from your own viewpoint. Also, if you feel uncomfortable about answering any of the questions, you do not have to answer them.

If you agreed, we will record our conversation, but the recording will only be heard by members of the research team. When we report our findings, any of your words/quotes will not have your real name attached, so no statement could be linked directly to you. This study is for the purposes of research only and aims to contribute new information that could help improve adherence to diabetes treatment.

Do you have any questions before we start? If not, you can ask anytime while we are talking and again at the end of the interview.

1. What is daily life with diabetes like? Could you tell us what are the most important things you would like to share with us about your experience of diabetes.

*Probes:*

1. What is your understanding of the diabetes as a disease and how it can best be managed?

(Explore level of health literacy: awareness of chronicity, of life-style components; prevention of complications; names of meds and dosages used)

1. What is your biggest challenge with managing the disease?
2. What has worked well for you in managing the disease?
3. Who knows you have diabetes and how does it matter?
4. How much do you feel you are in control of managing your disease?
5. Issue of their sense of well-being and/or the level of physical complications they may have experienced
6. How do you manage your diabetes treatment and medication?

*Probes:*

1. Do you take medication for other diseases? Which ones?
2. How do you manage that?
3. If you have had side-effects of meds, how have you dealt with this?
4. How do you manage accessing your medications on a monthly basis?
5. What if any has been the challenges with picking up meds?
6. What do you manage to remember to pick up the meds regularly? (get details of the reminder systems they use)
7. If you have a system, how well does your system for remembering / reminder system work?
8. Does it sometimes happen that you forget to pick up you meds? How often does that happen?
9. What happens when you forget to pick up your meds?
10. What happens when you run out of meds?
11. How do you remember to take your meds regularly? (get details of the reminder systems they use)
12. How well does that system work?
13. What else do you do to help you cope with the disease? (check areas of nutrition, physical activity, social support)
14. Where do you get your information from about diabetes and how to keep yourself healthy?
    - Sources, Type of info
    - Whose advice are you most likely to follow (about their medication and lifestyle)? And WHY?
15. How do you manage your relationship to the clinic staff? Reasons
    - *What are the most helpful things the clinic does?*
    - *What is less helpful? /stands in the way of a good relationship with the clinic/staff*
16. What kinds of support do you have for managing your diabetes? ( Check sources and types of support e.g. Family, community, health services/religion etc
17. Could you tell us about how you use your mobile phone?

*Probes:*

1. Own/Shared phone?
2. Can you show me your phone? (Get a sense of type of phone and its importance/value to the person)
3. What do you use the phone for?
   - (Calls? Messages?-explore more; Reminders?- explore more , Calendar?- explore more; Calculator?/Flash light?/Radio?/Web access/Facebook.check if WhatsApp used and how.
   - Check if this is used for social contact (discussion groups)
4. How did you learn to use various functions?
5. Which functions would you like to know how to use?
6. What do you not like about having a cell-phone?
7. How often is your phone switched on and with you?
8. Attitude to receiving SMS-texts?
9. What types of sms do you receive? (check if from bank, eskom, or hospital/clinic, advertisments, health care provider’s, SSASA)
10. What kind of SMS messages about your health care would you find helpful?
11. In which ways do you think your cellphone can help you with your health care/
12. How do you feel about receiving these different SMSses
13. How would you feel about receiving SMSses from you clinic about your health care?
14. What kind of SMS messages would be helpful?/Not helpful?
15. What specific sms communication about diabetes would be helpful? And Why?
16. What if a person has more than one illness- how do you think the SMS –messages should take this into account?
17. What would be the downside of receiving SMS reminders and health advice on your phone? (Checking for concerns re confidentiality, information overload etc.)
18. How often do you think these kinds of SMSses should be sent?

Expectations of StAR trial (for Pre-trial individual interview participants only)

1. What are your initial thoughts/expectations about the *StAR trial?
2. Why did you decide to sign-up for the study?
3. What is your understanding of the aim of the study?
4. And what do you know about what will happen next in this study, and in the next 12 months?
5. What do you hope to gain from participating in the study?
6. Do you think the SMSes you get from the *StAR2D study will fit/help with the reminder systems you currently have for taking your meds and collecting them from the clinic?
7. Do you have any concerns or worries about participating?

We are now wrapping up the discussion:

1. Do you have any final comments or questions?
2. Thank you so much for your time and your willingness to share your experience.
